# Impaired Urine Dilution Capability in HIV Stable Patients

**DOI:** 10.1155/2014/381985

**Published:** 2014-03-31

**Authors:** Waldo H. Belloso, Mariana de Paz Sierra, Matilde Navarro, Marisa L. Sanchez, Ariel G. Perelsztein, Carlos G. Musso

**Affiliations:** ^1^Infectious Diseases Section, Internal Medicine Service, Hospital Italiano de Buenos Aires, Peron 4190, 1181 ACH Buenos Aires, Argentina; ^2^Renal Physiology Section, Nephrology Service, Hospital Italiano de Buenos Aires, Peron 4190, 1181 ACH Buenos Aires, Argentina

## Abstract

Renal disease is a well-recognized complication among patients with HIV infection. Viral infection itself and the use of some antiretroviral drugs contribute to this condition. The thick ascending limb of Henle's loop (TALH) is the tubule segment where free water clearance is generated, determining along with glomerular filtration rate the kidney's ability to dilute urine. * Objective*. We analyzed the function of the proximal tubule and TALH in patients with HIV infection receiving or not tenofovir-containing antiretroviral treatment in comparison with healthy seronegative controls, by applying a tubular physiological test, hyposaline infusion test (Chaimowitz' test). * Material & Methods*. Chaimowitz' test was performed on 20 HIV positive volunteers who had normal renal functional parameters. The control group included 10 healthy volunteers. * Results*. After the test, both HIV groups had a significant reduction of serum sodium and osmolarity compared with the control group. Free water clearance was lower and urine osmolarity was higher in both HIV+ groups. Proximal tubular function was normal in both studied groups. * Conclusion*. The present study documented that proximal tubule sodium reabsorption was preserved while free water clearance and maximal urine dilution capability were reduced in stable HIV patients treated or not with tenofovir.

## 1. Introduction 

Renal disease is increasingly recognized as a cause of morbidity and mortality among patients with HIV infection [1]. Longer survival of patients on antiretroviral treatment as well as specific effects of some antiretroviral drugs and viral-related factors contributes to this finding [2].

Traditional risk factors for renal disease such as hypertension, diabetes, and hyperlipidemia are more prevalent in seropositive patients and are also associated with the advancing age of the HIV-positive population [3]. In addition, these variables can be associated with exposure to combined antiretroviral therapy (cART) which may induce direct toxic effects on the nephron [2]. Although the underlying mechanism is still unclear, tenofovir may produce proximal tubule damage characterized by proteinuria, normoglycemic glycosuria, hypokalemic renal tubular acidosis, and phosphaturia [4]. These relatively infrequent findings may appear in the context of preserved glomerular filtration rate (GFR), although in some studies a decrease in GFR has been reported [5–9].

In addition, proteinuria is an important finding in patients with HIV infection. Its prevalence in patients under cART ranges between 10 and 30% and constitutes an independent risk factor for progression of CKD and all-cause mortality [10–15].

Standard assessment of renal health includes usually GFR and proteinuria but has little focus on more specific markers of tubular function. Nevertheless, both drugs and HIV “per se” may produce tubular damage [16–20]. One of the main functions of the renal tubules is the reabsorption of substances and the determination of the amount of solute-compromised and solute-free water that will be excreted in urine. The evaluation of tubular function in routine clinical practice is somewhat more complex since it requires the analysis of fractional excretion of solutes and/or to perform specific tubular physiological tests [21, 22].

The objective of the present study was to analyze the function of the proximal tubule and the thick ascending limb of Henle's loop (TALH) in patients with HIV infection receiving or not tenofovir-containing antiretroviral treatment in comparison with seronegative controls, by applying a validated tubular physiological test known as “hyposaline infusion test” or Chaimowitz' test.

## 2. Materials and Methods

Patients with confirmed HIV infection were selected in accordance with prespecified criteria.

Inclusion criteria were as follows: adult patients (≥18 years old) with confirmed chronic HIV-1 infection who agreed to provide written informed consent. Patients under antiretroviral treatment must have a stable regimen for over six months and undetectable (<50 copies/mL) viral load for at least three months. At study entry, all selected patients were confirmed as having normal physical examination and routine clinical laboratory including urinalysis, as well as renal and cardiac ultrasound.

Exclusion criteria included patients with acute HIV infection (<6 months of disease) and personal history of nephropathy, plasma creatinine ≥1.3 mg/dL, GFR ≤ 60 mL/min/1.73 m² (as determined by MDRD formula), presence of glucosuria/proteinuria (measured in spot urine sample), prior heart failure, concurrent opportunistic infection, chronic active hepatitis B or C, and use of drugs which could potentially modify renal physiology in the prior week before the test (e.g., diuretics, cotrimoxazole, pyrimethamine, angiotensin converting enzyme inhibitors, AT1 angiotensin II receptor antagonists, or nonsteroidal anti-inflammatory agents).

A convenience group of age and gender-matched seronegative healthy volunteers was also selected for comparisons and applying same exclusion criteria as well as studying entry evaluation.

Patients with antiretroviral treatment were grouped in containing an non-containing tenofovir regimens.

Proximal and thick ascending limb of Henle's loop (TALH) tubular functions was evaluated by Chaimowitz' test in all volunteers (HIV tenofovir, HIV nontenofovir, and healthy people).

### 2.1. Chaimowitz' Test

Functional evaluation of the proximal tubule and the TALH was carried out using the Chaimowitz' test, which is based on the exploration of the tubular response to an acute fluid load [23, 24]. After overnight fast, all participants received twenty cc/Kg of mineral water per os and two liters of intravenous hypotonic solution (0.66%) infused in two hours. Three blood samples were drawn (at 0, 60, and 120 minutes) and also urine samples were collected from each person at baseline and at 30 (±5) minutes intervals during the whole test. From the obtained blood and urine samples, glucose, urea, creatinine, and osmolarity were measured, and then from the data corresponding to the most hypotonic urine sample (maximum dilution) and its corresponding blood sample three renal physiological parameters (proximal sodium clearance, free water clearance, and sodium TALH reabsorption) were analyzed by applying the following formulas:

- V = urine volume (mL/min);
- osmolal clearance = urinary osmolarity × V/serum osmolaity (mL/min/1.73 m²);
- free water clearance = V − osmolal clearance (mL/min/1.73 m²);
- sodium clearance = V × urinary sodium/serum sodium (mL/min/1.73 m²);
- potassium clearance = V × urinary potassium/serum potassium (mL/min/1.73 m²);
- sodium + potassium clearance = [(urinary sodium + urinary potassium) × V]/(serum sodium + potassium) (mL/min/1.73 m²);
- proximal tubule function = free water clearance + sodium clearance + potassium clearance (mL/min/1.73 m²);
- TALH sodium reabsorption = free water clearance × 100/free water clearance + sodium clearance (%).

The salt and water load infused during this test suppresses the activity of antidiuretic hormone (ADH) and aldosterone and thus “defunctionalizes” the collecting tubules. Sodium is the major solute reabsorbed at the proximal tubule, and thus the function of this segment can be analyzed through its local sodium clearance. In contrast, water handling may account for the evaluation of the TALH, since this segment is responsible for the generation of free water clearance (or local sodium reabsorption) which in turn contributes to medullar hypertonicity and consequently to water reabsorption capability in the collecting tubules under water restriction.

As previously demonstrated, the presence of renal incompetence for sodium reabsorption in the proximal and TALH segments can be detected by the Chaimowitz' test [25].

In addition, since electrolytes (potassium, magnesium, calcium, and phosphorus) were also measured in all samples, the analysis of the fractional excretion (FE) of phosphorus and uric acid—mainly reabsorbed at the proximal segment—was obtained as an alternative way of assessing proximal tubule functioning. Similarly, the analysis of the FE of magnesium—mainly reabsorbed in the TALH—was obtained as an alternative way of evaluating this tubular segment.

The present study was approved by the Institutional Review Board and all participants provided written informed consent prior to the performance of all the study evaluations.

Comparisons between both of the groups were performed with Mann-Whitney Wilcoxon Rank Sum Test and among three groups with Kruskal-Wallis Test. Comparisons within groups pre- and postdilution were performed with Wilcoxon Signed Rank Test, with *P* < 0.05 as the level of significance.

## 3. Results

A total of 30 patients were included in the analysis. Among patients with HIV infection, ten individuals (1 female) were currently receiving stable antiretroviral treatment regimen including tenofovir (tenofovir group), while eleven patients (1 female) were not receiving tenofovir (nontenofovir group)—in fact three patients were not receiving antiretroviral treatment. Median CD4+ T-cell count was 464 cells/mm^3^ (r280–869) and 475 cells/mm^3^ (r293–1876) in the tenofovir and nontenofovir groups, respectively. Nine seronegative healthy volunteers (all males) were selected as control. Median age was 48 (42–71) and 51 (20–71) years in both HIV positive groups and 46 (28–50) years in the control group.

Baseline (pretest) serum urea, calcium, sodium, and magnesium values showed a significant reduction compared to their same values underwent a significant reduction at maximal dilution status in both HIV groups (Table 1). However, basal serum potassium and phosphorus did not show a significant reduction at maximal dilution status in HIV tenofovir group, while they did in the HIV nontenofovir one (Table 1). Most of FE (sodium, chloride, phosphate, urea, and uric acid) showed no significant difference between their basal and dilution values in both groups, except for FE of potassium and magnesium in the HIV nontenofovir group and FE of calcium in both HIV groups which showed a higher value in the dilution status (Table 2).

Most serum and FE documented values were within normal range in both HIV groups, except for maximum dilution plasma osmolarity (PO) and urine osmolarity (UO) which were lower and higher, respectively, than the expected one (Table 3).

Mild (asymptomatic) hypokalemia (e.g., 2.7 mmol/L), hypocalcemia (e.g., 7.7 mg/dL), and hypomagnesemia (e.g., 1.5 mmol/L) were documented in some HIV patients (both groups) at maximal dilution status.

Although no significant difference was observed between both HIV groups, they showed significantly lower free water clearance (normal: 10–18 mL/min/1.73 m²) and lower sodium TALH reabsorption (normal: ≥80%) than those observed in the healthy volunteer group (control group) (Table 4). Multivariate analysis found no significant difference in Chaimowitz' test results among HIV patients despite their treatment: tenofovir (n: 10), nontenofovir cART (n: 8), or without cART (n: 3).

## 4. Discussion

During this study most baseline (fasting) serum and fractional excretion values of electrolytes were similar between HIV groups and—as expected—were within normal range (Table 1). Analyzing the values obtained with acute volume load we point out the following interesting findings.

1. Proximal tubule sodium clearance and indirect proximal tubule function markers such as FE of phosphorus and FE of uric acid showed normal values at basal and hyposaline load status in both HIV groups.
2. TALH functional markers (free water clearance and TALH sodium reabsorption) showed abnormally low values in both HIV groups. This phenomenon could explain why some HIV patients (in both groups) were not able to reduce urine osmolarity below the expected value (<100 mOsm/L) along the Chaimowitz' test (Table 3).

Healthy kidney has an enormous capacity to excrete free water, and this capacity depends on the following physiological variables:

- an adequate GFR, since it delivers urine to the diluting segment (TALH),
- a preserved TALH function (where free water clearance is generated), and
- an impermeable collecting tubules (absence of vasopressin).

Thus, a patient suffering from a severely reduced GFR (<10 mL/min) and/or a critically low free water clearance (<5 mL/min) can easily develop a free water body excess (hypoosmolar hyponatremia) in a context of a high water supply [26–28].

In our study patients with HIV infection showed a markedly reduced free water clearance and urine dilution capability (Table 3). Free water clearance impairment was more pronounced in both HIV groups, where it showed values three times lower than the normal one. This could partially explain why HIV patients, despite their normal GFR, were not able to maximally dilute their urine during hyposaline infusion test (water load: 1700 cc/hour) or able to avoid developing hyponatremia during this physiological test. Healthy people either young or old, do not develop hyponatremia during hyposaline infusion test since they are able to adequately dilute their urine, meaning to achieve a UO lower than 100 mOsm/L.

It could be argued that hyponatremia developed by HIV patients during hyposaline test could be secondary to an inappropriate (nonosmolar) antidiuretic hormone release (SIADH). However, there are some findings against this interpretation such as the fact that HIV patients showed neither basal hyponatremia (Table 2) nor increased FE of sodium (FE > 1%) (Table 2) nor high urine osmolarity (UO > 300 mOsml/L) during the hyposaline infusion test (Table 3); although other findings are not against the hypothesis of inadequate antidiuretic hormone release or excessive kidney response to this hormone, such as the documented high basal FE of urea (Table 2) or the observed incapability of volunteers to reduce urine osmolarity below 100 mOsm/L at maximal dilution status (Table 4).

Additionally, it is well established that fluid load increases urine tubular flux and consequently can lead to an increase in electrolyte urine excretion, as well to a slight reduction in their serum values. This phenomenon could explain the reduced serum electrolytes levels and the increased FE of solutes documented during hyposaline infusion in both HIV groups. Regarding the mild hypokalemia, hypocalcemia, and hypomagnesemia documented in some HIV volunteers at dilutional status, this could be explained by the observed TALH dysfunction, given the important role that this segment plays in handling these electrolytes.

It has already been described in the literature that hyponatremia may occur in up to 56% of hospitalized patients with acquired immunodeficiency syndrome (AIDS) patients, a higher prevalence for hyponatremia than the one expected in the rest of hospitalized population, 15%. Thus, hospitalized patients with AIDS are at higher risk of developing hyponatremia than the general patient group. This hyponatremia has been attributed to several factors, such as the use of hyponatremia inducing drugs (opiates, etc.), active pulmonary diseases (Pneumocystis jirovecii pneumonia, etc.), and central nervous system lesions (CMV encephalitis, etc.). Conversely, in our study HIV volunteers had no AIDS, no concomitant diseases, nor medication which represented hyponatremia inducing factor. Thus, our original findings suggest that there would be an intrinsic incapability to maximally dilute urine in stable HIV patients [29, 30].

In this protocol, at study entry all participants had normal baseline routine plasma analysis, urinalysis, and renal ultrasound in order to exclude the presence of CKD which may itself modify free water clearance and consequently urine dilution capability. This could explain why a normal proximal tubule function was observed in the studied HIV volunteers either receiving or not antiretroviral drugs. Additionally, the mild hyponatremia developed by volunteers during hyposaline infusion test barely be attributed to any of the used antiretroviral drugs (tenofovir-lamivudine-abacavir-efavirenz) since to our knowledge there is no report in the literature which has related this adverse effect to them [26–28, 31].

Our original findings suggest that kidney's ability to dilute urine is impaired in stable patients with HIV infection (treated or not with tenofovir), with the potential risk to develop osmolality abnormalities under water load scenarios. HIV itself has been detected in tubular cells suggesting that either the infection or the associated inflammatory process may produce a direct tubular damage which appears to be independent of the presence of antiretroviral treatment.

In the clinical setting, these findings indicate an increased risk for hyponatremia even in stable HIV-infected patients undergoing water load as well as in the context of receiving drugs that could cause hyponatremia such as diuretics or psychoactive drugs.

## 5. Conclusion

In this study it was documented that proximal tubule sodium reabsorption was preserved while free water clearance and maximal urine dilution capability were reduced in stable HIV patients treated or not with tenofovir suggesting an impairment in TALH function associated with HIV infection.

## 6. Limitations

Although extremely uniform in our study, our finding may require further confirmation given the small number of patients evaluated, in particular patients without antiretroviral treatment.

In addition, the low representation of women precludes a gender wise generalization of our results.

An additional limitation of our study is that serum vasopressin levels were not measured, although conventional Chaimowitz' test does not require that. Besides, serum normal vasopressin level does not exclude SIADH (syndrome of inappropriate antidiuretic hormone secretion) since a sort of SIADH which runs with normal serum level of this hormone (nephrogenic SIADH) has already been reported [32].

## Figures and Tables

**Table 1:** Comparison of serum levels of solutes between HIV tenofovir group (T) and HIV nontenofovir group (nT) at baseline (B) and at maximum dilution (D) (Chaimowitz' test): serum values—median and range.

	HIV tenofovir baseline	HIV tenofovir dilution	*P* value	HIV nontenofovir baseline	HIV nontenofovir dilution	*P* value	*P* value T (B) versus nT (B) T (D) versus nT (D)
Serum urea (mg/dL)	32.5 (14–53)	26 (14–44)	0.01	36 (15–57)	33 (12–50)	0.003	NS
Serum creatinine (mg/dL)	0.82 (0.65–1.2)	0.81 (0.65–1.1)	NS	0.85 (0.58–1.07)	0.84 (0.52–1.0)	NS	NS
Serum glucose (mg/dL)	84 (68–99)	106.5 (75–145)	0.01	81 (69–105)	156 (60–220)	NS	NS
Serum uric acid (mg/dL)	4.8 (3.9–7)	4.2 (3.9–5.8)	NS	4.9 (3.1–5.4)	4.2 (2.8–6.6)	NS	NS
Serum calcium (mg/dL)	9.1 (8.1–10)	8.1 (7.7–8.8)	0.01	9.05 (8.8–10.4)	8.5 (7.8–8.7)	0.01	NS
Serum sodium (mmol/L)	137.5 (136–141)	134 (131–137)	0.009	136 (135–143)	133 (129–140)	0.01	NS
Serum potassium (mmol/L)	3.95 (3.7–5.2)	3.7 (2.7–5.0)	NS	4 (3.6–5.6)	3.6 (2.9–4.3)	0.005	NS
Serum phosphate (mmol/L)	4.1 (2.3–4.8)	2.9 (2.1–3.9)	NS	3.45 (3.2–4.3)	2.85 (2.1–3.2)	0.01	NS
Serum magnesium (mmol/L)	2.25 (2.0–2.4)	2.0 (1.8–2.2)	0.02	2.05 (1.9–2.2)	1.8 (1.5–2.0)	0.01	0.03
Serum chloride (mmol/L)	104 (96–107)	104.5 (96–108)	NS	103 (100–107)	105 (99–107)	NS	NS

**Table 2:** Comparison between HIV tenofovir group (T) and HIV nontenofovir group (nT) at baseline (B) and at maximum dilution (D) (Chaimowitz' test): eGFR, fractional excretion (FE), and plasma and urine osmolarity values—median and range.

	HIV tenofovir baseline	HIV tenofovir dilution	*P* value	HIV nontenofovir baseline	HIV nontenofovir dilution	*P* value	*P* value T (B) versus nT (B) T (D) versus nT (D)
eGFR (MDRD) (mL/min/1.73 m^2^)	103.9 (65–125)	103.9 (72–134)	NS	98 (79.4–139)	100 (79.4–141)	NS	NS
FE sodium (%)	0.59 (0.25–1.8)	0.78 (0.48–1.27)	NS	0.52 (0.4–1.0)	0.87 (0.36–1.56)	0.01	NS
FE potassium (%)	4.9 (3.3–11.1)	7.8 (1.18–19)	NS	6.4 (0.8–13)	13.3 (3.5–18)	0.005	0.04
FE chloride (%)	0.68 (0.47–3.7)	1.4 (1.1–1.7)	NS	0.58 (0.28–5.7)	1.6 (0.81–5.0)	NS	NS
FE phosphate (%)	14 (6–20)	15.6 (3.79–24)	NS	15.5 (3–23)	10.75 (0.6–19)	NS	NS
FE calcium (%)	0.68 (0.34–1.5)	1.4 (0.99–1.7)	0.02	0.58 (0.46–1.3)	2.0 (0.19–2.8)	0.02	0.04
FE magnesium (%)	2.05 (0.8–3)	2.7 (2.2–7.48)	NS	1.53 (0.44–4.9)	4.2 (0.45–5.0)	0.04	NS
FE urea (%)	47 (21–60)	46 (27–300)	NS	57 (20–460)	65 (44–110)	NS	NS
FE uric acid (%)	4.7 (4.1–7.9)	7.9 (6.0–10.0)	NS	4.4 (0.36–8.7)	10 (2.0–29)	NS	NS
Plasma osmolarity (mOsm/L)	282.5 (279–292)	278.5 (270–283)	0.009	285 (277–298)	278 (273–289)	0.005	NS
Urine osmolarity (mOsm/L)	418.5 (142–759)	138.5 (38–594)	0.005	659 (369–854)	92 (37–323)	0.003	NS

**Table 3:** Plasma and urine osmolarity values in HIV groups.

	HIV tenofovir	HIV nontenofovir	Seronegative controls
Fasting UO (mOsm/L)	418.5 (142–759)	659 (369–854)	940 (860–1010)
Maximum dilution PO (mOsm/L)	278.5 (270–283)	278 (273–289)	286 (281–290)
Maximum dilution UO (mOsm/L)	138.5 (38–594)	92 (37–323)	59 (31–62)

UO: urine osmolarity, PO: plasma osmolarity.

**Table 4:** Chaimowitz' test specific results. Comparison between HIV groups and seronegative controls: median (range) values.

	HIV tenofovir group	HIV nontenofovir group	Seronegative controls	*P* value
Urine osmolarity (mOsm/L)	138.5 (38–594)	92 (37–323)	59 (31–62)	0.01
Free-water clearance (TALH function) (mL/min/1.73 m^2^)	3.07 (−1.36–6.9)	5.5 (−0.42–8.6)	13.2 (10.1–18.2)	<0.001
Proximal sodium clearance (proximal tubular function) (mL/min/1.73 m^2^)	1.15 (0.59–4.1)	1.4 (0.47–2.2)	13.3 (10.8–19.9)	0.0001
Distal sodium reabsorption (Henle) (%)	71 (18–93)	91 (61–98)	84 (81–90)	0.04
Osmolar clearance (mL/min/1.73 m^2^)	2.17 (1.0–6.3)	2.6 (0.78–3.39)	2.4 (1.9–3.9)	0.005

TALH: thick ascending limb of the loop of Henle.
